# Laboratory evaluation of the speed of kill of lotilaner (Credelio™) against *Ixodes ricinus* ticks on dogs

**DOI:** 10.1186/s13071-017-2467-z

**Published:** 2017-11-01

**Authors:** Martin Murphy, Daniela Cavalleri, Wolfgang Seewald, Jason Drake, Steve Nanchen

**Affiliations:** 1 Elanco Animal Health, Mattenstrasse 24a, 4058 Basel, Switzerland; 2 Elanco Animal Health, 2500 Innovation Way, Greenfield, IN 46140 USA

**Keywords:** Lotilaner, Credelio, Ticks, *Ixodes ricinus*

## Abstract

**Background:**

With the geographical expansion of tick species and increased recognition of pathogens they transmit, there is a requirement for safe and rapidly effective control measures for dogs. Lotilaner, a novel isoxazoline, is rapidly absorbed following administration of a flavored chewable tablet formulation (Credelio™), providing at least 98% efficacy for at least 1 month following assessments at 48 h post-treatment, and following subsequent challenges. A study was conducted to determine the speed with which lotilaner kills ticks.

**Methods:**

From 38 dogs, the 32 with the highest *Ixodes ricinus* counts from a Day -4 infestation were randomized among four groups: two groups were untreated controls, two received lotilaner tablets at a minimum dose rate of 20 mg/kg. Infestations with *I. ricinus* were performed on Days -2, 7, 14, 21, 28 and 35. Counts were completed 4 and 8 h post-treatment (Day 0), and 8 and 12 h following subsequent infestations. All live ticks were incubated for 24 h following removal from study dogs.

**Results:**

At 4 h post-treatment, there was a 69.8% reduction in geometric mean live tick counts in treated dogs compared to controls. After incubation, the reduction increased to 97.2%. At 8 h after treatment, pre- and post-incubation reductions were 99.2 and 100%, respectively. Following post-treatment challenges, post-incubation efficacy through Day 28 at 8 and 12 h was at least 94.3 and 98.0%, respectively, and was 85.7 and 94.2% at 8 and 12 h after the Day 35 challenge. Mean live tick counts in the lotilaner groups were significantly lower than in the control groups at all assessments through Day 35 at 8 (*t*_(7)_ ≥ 9, *P* < 0.0001, Days 0 to 28; *t*_(7)_ = 3.54, *P* ≤ 0.0095, Day 35) and 12 h post-treatment and after subsequent infestations (*t*_(7)_ ≥ 10, *P* < 0.0001, all days). There were no treatment-related adverse events.

**Conclusion:**

Lotilaner at a minimum dose rate of 20 mg/kg began to kill ticks on dogs within 4 h of treatment and efficacy was 100% within 8 h. Lotilaner sustained a rapid kill of newly infesting *I. ricinus* through 35 days. By quickly killing ticks that infest dogs, lotilaner has potential to help limit the transmission of tick-borne pathogens.

**Electronic supplementary material:**

The online version of this article (10.1186/s13071-017-2467-z) contains supplementary material, which is available to authorized users.

## Background

Since the first identification of *Borrelia burgdorferi* as the causative agent of Lyme disease in the 1980s the number of recognized, medically significant tick-borne diseases has increased dramatically [1]. The geographical expansion of tick species and growing incidence of diseases caused by the pathogens they transmit has been attributed to man-made changes in land use, to socio-economic changes with the expansion of human habitats into new areas such as woodlands, to wildlife migration, and to the international transport of animals [2–5].

The discovery of a novel family of compounds, the isoxazolines, is providing a valuable addition to the measures that are available for the management of tick infestations, and for reducing the risk of dogs contracting tick-borne diseases [6–8]. When administered orally to dogs, the isoxazolines offer a means of tick control that does not require the owner to carefully apply a product directly onto the dog’s skin to ensure optimal effectiveness. Other benefits of orally administered products compared to those that are topically applied link to the absence of concerns of reduced effectiveness with varying climatic conditions, of exposure of treated animals to water during swimming or bathing, and of potential pesticide exposure of household family members [9, 10].

Lotilaner is a novel isoxazoline that is rapidly absorbed following oral administration to dogs. Following treatment, insecticidal and acaricidal activity is then maintained for at least 30 days [11–13]. Studies completed for registration showed that against *Ixodes scapularis*, *Ixodes ricinus*, *Dermacentor variabilis*, *Dermacentor reticulatus*, *Rhipicephalus sanguineus* and *Amblyomma americanum*, lotilaner eliminated 100% of tick burdens 48 h after treatment of existing infestations [12]. Lotilaner effectiveness of at least 98% was sustained against each of those species at 48 h after weekly challenges for at least 4 weeks. Nonetheless, by 48 h post-infestation, ticks may have sufficient time to transfer pathogens to a host, with the risk of transfer increasing from the time the tick first begins to attach [14–16]. There was therefore a need to determine lotilaner’s sustained speed of kill (SOK) against new tick challenges throughout the product protection period of at least 1 month. To address this need a study was designed with the primary objective of determining the SOK of lotilaner flavored chewable tablets following a single oral administration, at a minimum dose rate of 20 mg/kg.

## Methods

This blinded, randomised, parallel-group laboratory study was carried out in accordance with the protocol and in compliance with the VICH guideline on Good Clinical Practice (GCP; VICH GL 9), and in compliance with the relevant national legislation [17]. The protocol was approved by the Ethics Committee of the laboratory.

### Animals and housing

Thirty-eight healthy Beagle dogs from the laboratory’s colony were acclimated to study conditions from 1 week prior to treatment. For inclusion dogs were required to be healthy, to be older than 7 months at the initiation of the acclimation period and to have had a live tick attachment rate of at least 25% of applied female ticks from an infestation applied on Day -4. Dogs were excluded if during the previous 60 days they had been involved in any study or were treated with any compounds having activity against ticks. Dogs were also excluded if they had been treated with any isoxazoline compounds within the prior 6 months. Selected dogs ranged in age from 15 to 85 months and weighed from 10.7 to 17.5 kg. To facilitate tick counting and tick infestation, each dog was individually housed before infesting with ticks. Between tick challenges, dogs were pair-housed in caged concrete-floored pens. For the duration of the study, the temperature remained between 17 and 19 °C and the humidity ranged between 42 and 69%. Lighting was controlled to give approximately 10 h light and 14 h darkness per 24 h period. When tick counting occurred during the 14-h dark period the lights were turned on prior to the start of the tick count and turned off after the tick count was completed. Standard commercially available dog food was fed at the recommended rates from Study Day -7 to Study Day 36. Potable water was available ad libitum *via* stainless steel drinkers.

### Randomization and treatment

Dogs that met all the inclusion criteria and that had none of the exclusion criteria were ranked in descending order of Day -4 tick counts (48 h after infestations). The 32 dogs (16 male, 16 female) that had the highest counts and at least a 25% attachment rate were randomly allocated to four groups of eight dogs per group.

Within sex, the animals were ranked based on highest to lowest tick count (where more than one animal had the same count, they were ranked in order of decreasing animal identification number). The first four males formed a block, the next four males formed a second block and so on until four blocks of four males had been formed, and the same process was followed for females. Animals within a block were then assigned to the four study groups using random order numbers derived from Fisher and Yates tables.

Groups 1 and 2 were mock-dosed untreated controls. Groups 3 and 4 were treated on Day 0 with lotilaner flavored tablets at as close as possible to the minimum dose rate of 20 mg/kg. All dogs were fed within approximately 30 min prior to treatment. Each dog was observed for successful intake of the dose immediately after administration, at 30 min (± 5 min) and at 1 h (± 10 min) after administration. No vomit was present for any animal at any of the checks post dosing.

### Tick infestations and counts

For randomization and efficacy assessments, dogs were infested with 50 ± 4 adult *I*. *ricinus* ticks (approximate sex ratio 60% female: 40% male). Dogs were sedated prior to application of the ticks by intramuscular injection of 0.04 ml/kg medetomidine hydrochloride (1 mg/ml) which, after infestations, was reversed by intramuscular injection of atipamezole hydrochloride (5 mg/ml) at a dose rate of 0.04 ml/kg. Tick infestations were completed on Days -6, -2, 7, and then weekly through Day 35. Vials with the correct number of ticks were shaken until none were clinging to the container. The ticks were then quickly shaken onto the lumbosacral region of the sedated dogs.

Tick counts were completed on Day -4 (48 h post infestation), Day 0, 4 h (+10 min) (Groups 1 and 3) and 8 h (+15 min) (Groups 2 and 4) after treatment, and on Days 7, 14, 21, 28 and 35 at 8 h (+15 min) (Groups 1 and 3) and 12 h (+ 1 h) (Groups 2 and 4) after infestation. Ticks were removed carefully, using a removal device, to ensure they were not damaged.

The numbers of live attached and live free ticks on the dogs were counted. Since male *I. ricinus* ticks do not attach, they were not included in the count and were discarded in methylated spirits. Ticks were considered alive if legs reacted to a tactile or exhaled air (CO_2_) stimulus and were considered dead if they did not. Dogs were not sedated for tick counts. To ensure that all ticks had been located, personnel checked each dog’s body very carefully, moving the fur against the grain. Examination time was at least 5 min. Following tick removal, all animals were combed to ensure no further ticks were present.

All live attached and live free ticks removed from the dogs were counted and placed into containers, each with a vented cap, which were immediately transferred to an incubator at 26.1–28.8 °C and 74–88% relative humidity. The viability of the ticks and their engorgement status was assessed 24 h (+2 h) after removal from each dog.

### Assessment of efficacy

Efficacy was defined at each post treatment assessment as a reduction of greater than 90% in mean live tick counts in each treated group, compared to the corresponding control group. Geometric and arithmetic and means were calculated of live (live free and live attached) ticks 24 h after incubation. Efficacy was calculated as follows:

$$ \mathrm{Percent}\  \mathrm{effectiveness}=100\times \left(\left(\mathrm{C}\hbox{--} \mathrm{T}\right)/\mathrm{C}\right) $$

where: C is the mean number of live (live free and live attached) ticks in the control group and T is the mean number of live (live free and live attached) parasites in the treated group.

Since the calculation of the geometric mean involved taking the logarithm of the parasite count of each dog, when any tick counts were equal to zero, a one was added to the count for every animal in every treatment group and then subtracted from the resultant calculated geometric mean prior to calculating percent effectiveness. For the relevant tick counts on a given day, an ANOVA model was used to compare treated and untreated groups. The model was applied to the log-transformed counts. There was one fixed effect, treatment group; and one random effect block. This method was applied pre- and post-incubation of ticks. The significance of the treatment effect was then assessed with a *t*-test. Tick counts before and after incubation were compared in a paired-sample *t*-test. All calculations were carried out using the software SAS/STAT®, Version 9.2.2.

Infestations were considered adequate at each measurement day/h if at least 25% on average of the applied female *I. ricinus* ticks were attached to control dogs. Efficacy was achieved if in the ANOVA there was a significant difference between tick counts of the treated and control groups on the two-sided 5% level of significance, and if the treated group had a percent efficacy of more than 90%.

### Safety assessment

The general health of all dogs was observed by a trained technician once daily except on Day 0 when clinical observations were performed. Clinical observations included a physical examination and assessments of body weights, behavior, salivation, pupillary constriction, nervous signs, and presence and consistency of feces. These observations were performed on each dog prior to treatment and at 1 h (± 10 min), 6 and 8 h (± 30 min) after treatment. All concomitant medications were administered following the recommendations of a licensed veterinarian.

### Translation

French translation of the Abstract is available in Additional file 1.

## Results

**Fig. 1:**
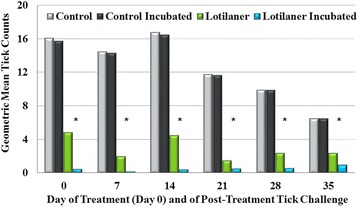
Geometric mean tick counts of Group 1 (control) and Group 3 (lotilaner-treated) before and after 24-h incubation on Day 0 (4 h post-treatment) and at 8 h after each subsequent infestation with *Ixodes ricinus*. Post-incubation means of treated group were significantly decreased in comparison to the untreated group (*t*_(7)_ ≥ 9, *P* < 0.0001 on all days except Day 35 when *t*_(7)_ = 3.54, *P* = 0.0095). *Pre- and post-incubation differences in mean counts in the lotilaner group were significant (*t*_(7)_ ≥ 2.64, *P* ≤ 0.0333)

**Fig. 2:**
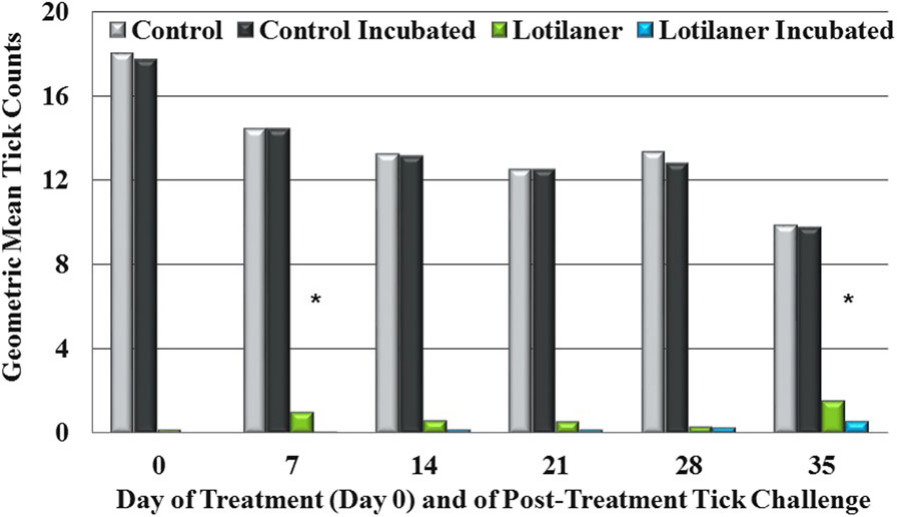
Geometric mean tick counts of Group 2 (control) and Group 4 (lotilaner-treated) before and after 24-h incubation on Day 0 (8 h post-treatment) and at 12 h after each subsequent infestation with *Ixodes ricinus*. Post-incubation means of treated group were significantly decreased in comparison to the untreated group (*t*_(7)_ ≥ 10, *P* < 0.0001 on all days). *Pre- and post-incubation differences in mean counts in the lotilaner group were significant (*t*_(7)_ ≥ 2.45, *P* ≤ 0.0440)

The arithmetic mean attachment rate of *I. ricinus* in the untreated control groups met the criteria of at least 25% at all assessment points (Table 1). No live free ticks were found at any assessment. There was no significant difference (*t*_(7)_ ≤ 1.7, *P* ≥ 0.13) in mean live tick counts before or after incubation for the control groups at any time point (Figs. 1 and 2). In the treated Group 3, on all study days geometric mean tick counts were significantly lower (*t*_(7)_ ≥ 2.64, *P* ≤ 0.0333) after incubation than before incubation. In the treated Group 4, geometric mean counts after incubation were significantly lower than before incubation only on Days 7 and 35 (*t*_(7)_ ≥ 2.45, *P* ≤ 0.0440), since on other days pre-incubation counts were already low (Fig. 2).

**Table 1:** Mean attachment rate of *Ixodes ricinus* ticks in untreated control groups at each study assessment

		Day of study	Mean infestation rate (%)
Group 1^a^	Tick count 4 h after treatment, before incubation	0	55.4
	Tick count 8 h after infestations, before incubation	7	50.8
		14	56.3
		21	40.4
		28	35.4
		35	29.2
Group 2^b^	Tick count 8 h after treatment, before incubation	0	62.9
	Tick count 12 h after infestations, before incubation	7	49.6
		14	45.4
		21	42.9
		28	45.8
		35	34.6

^a^For Group 1, 4-h counts were completed only on Day 0, and 8-h counts were from 7 to 35 days

^b^ For Group 2, 8-h counts were completed only on Day 0, and 12-h counts were from 7 to 35 days

**Fig. 3:**
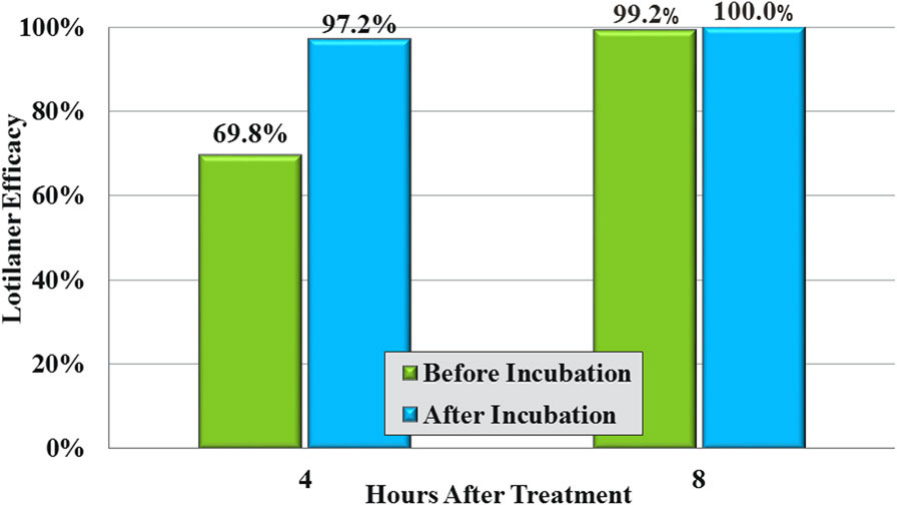
Day 0% reduction in geometric mean *Ixodes ricinus* counts of lotilaner-treated dogs compared to untreated control dogs

Compared to the corresponding control group, geometric mean counts were significantly lower for Group 3 (assessments at 4 h post-treatment, and at 8 h after all subsequent infestations) (*t*_(7)_ = 3.54, *P* = 0.0095, for Day 35; *t*_(7)_ ≥ 9, *P* < 0.0001, for Days 0 to 28) (Tables 2 and 3). At 4 h post-treatment, there was a 69.8% reduction in mean live tick counts in lotilaner-treated dogs compared to controls, and after the live ticks from each group had been incubated the reduction in mean live tick counts increased to 97.2% (Fig. 3). At 8 h post-treatment, the pre- and post-incubation reductions in mean live tick counts were 99.2 and 100%, respectively. Geometric mean live tick counts in the lotilaner groups were significantly lower than in the control groups at each weekly post-challenge assessment through Day 35 at 8 h (*t*_(7)_ = 3.54, *P* = 0.0095, for Day 35; *t*_(7)_ ≥ 9, *P* < 0.0001, for Days 7 to 28) and 12 h (*t*_(7)_ ≥ 10, *P* < 0.0001, for all days) post-infestation (Tables 3 and 4).

**Table 2:** Geometric (arithmetic) mean counts of *Ixodes ricinus* and percent efficacy of lotilaner against infestations present at the time of treatment

Time of count post-treatment		Untreated control		Lotilaner			Comparison
		Mean (arithmetic)	Range	Mean (arithmetic)	Range	Efficacy (%)	
4 h	Before incubation	16.0 (16.6)	10–23	4.8 (9.0)	0–29	69.8 (45.9)	*t*_(7)_ = 2.36, *P* = 0.0501
Group 1 (control) and Group 3 (lotilaner)	After incubation	15.7 (16.3)	10–23	0.4 (0.9)	0–5	97.2 (94.6)	*t*_(7)_ = 9.26, *P* < 0.0001
8 h	Before incubation	18.0 (18.9)	10–26	0.2 (0.3)	0–2	99.2 (98.7)	*t*_(7)_ = 15.43, *P* < 0.0001
Group 2 (control) and Group 4 (lotilaner)	After incubation	17.7 (18.6)	10–26	0.0 (0.0)	0–0	100 (100)	*t*_(7)_ = 24.61, *P* < 0.0001

**Table 3:** Geometric (arithmetic) mean counts of *Ixodes ricinus* and percent efficacy of lotilaner at 8 h following post-treatment challenge infestations (Group 1, control and Group 3, lotilaner)

Day of challenge		Untreated control		Lotilaner			Comparison
		Mean (arithmetic)	Range	Mean (arithmetic)	Range	Efficacy (%) (arithmetic mean efficacy)	
7	Before incubation	14.4 (15.3)	8–28	2.0 (2.8)	0–8	86.1 (82.0)	*t*_(7)_ = 6.76, *P* = 0.0003
	After incubation	14.3 (15.1)	8–28	0.2 (0.3)	0–1	98.7 (98.3)	t_(7)_ = 15.55, *P* < 0.0001
14	Before incubation	16.7 (16.9)	12–21	4.5 (6.0)	0–11	73.2 (64.4)	t_(7)_ = 3.78, *P* = 0.0069
	After incubation	16.4 (16.6)	12–21	0.4 (0.6)	0–3	97.5 (96.2)	t_(7)_ = 12.89, *P* < 0.0001
21	Before incubation	11.7 (12.1)	7–16	1.5 (2.0)	0–5	87.3 (83.5)	t_(7)_ = 6.23, *P* = 0.0004
	After incubation	11.6 (12.0)	7–15	0.5 (0.6)	0–2	95.8 (94.8)	t_(7)_ = 11.55, *P* < 0.0001
28	Before incubation	9.9 (10.6)	4–16	2.4 (3.4)	0–7	75.9 (68.2)	t_(7)_ = 4.55, *P* = 0.0026
	After incubation	9.9 (10.6)	4–16	0.6 (0.8)	0–2	94.3 (92.9)	t_(7)_ = 11.52, *P* < 0.0001
35	Before incubation	6.5 (8.8)	0–23	2.4 (3.3)	0–11	63.4 (62.9)	t_(7)_ = 2.25, *P* = 0.0590
	After incubation	6.5 (8.6)	0–22	0.9 (1.3)	0–3	85.7 (85.5)	t_(7)_ = 3.54, *P* = 0.0095

**Table 4:** Geometric (arithmetic) mean counts of *Ixodes ricinus* and percent efficacy of lotilaner at 12 h following post-treatment challenge infestations (Group 2, control and Group 4, lotilaner)

Day of challenge		Untreated control		Lotilaner			Comparison
		Mean (arithmetic)	Range	Mean (arithmetic)	Range	Efficacy (%) (arithmetic mean efficacy)	
7	Before incubation	14.4 (14.9)	10–23	1.0 (1.9)	0–9	93.2 (87.4)	*t*_(7)_ = 6.47, *P* = 0.0003
	After incubation	14.4 (14.9)	10–23	0.1 (0.1)	0–1	99.4 (99.2)	*t*_(7)_ = 20.98, *P* < 0.0001
14	Before incubation	13.2 (13.6)	9–17	0.6 (1.5)	0–6	95.3 (89.0)	*t*_(7)_ = 6.55, *P* = 0.0003
	After incubation	13.1 (13.5)	9–17	0.2 (0.3)	0–1	98.6 (98.1)	*t*_(7)_ = 17.21, *P* < 0.0001
21	Before incubation	12.5 (12.9)	8–18	0.6 (1.3)	0–5	95.5 (90.3)	*t*_(7)_ = 7.99, *P* < 0.0001
	After incubation	12.5 (12.9)	8–18	0.2 (0.3)	0–1	98.5 (98.1)	*t*_(7)_ = 21.49, *P* < 0.0001
28	Before incubation	13.3 (13.8)	8–20	0.3 (0.6)	0–4	97.5 (95.5)	*t*_(7)_ = 10.49, *P* < 0.0001
	After incubation	12.8 (13.3)	8–20	0.3 (0.4)	0–2	98.0 (97.2)	*t*_(7)_ = 13.28, *P* < 0.0001
35	Before incubation	9.9 (10.4)	5–17	1.5 (1.9)	0–4	84.6 (81.9)	*t*_(7)_ = 6.03, *P* = 0.0005
	After incubation	9.8 (10.3)	5–16	0.6 (0.8)	0–2	94.2 (92.7)	*t*_(7)_ = 10.59, *P* < 0.0001

**Fig. 4:**
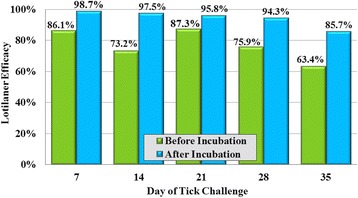
Percent reduction in geometric mean tick counts of lotilaner-treated dogs compared to untreated control dogs at 8 h after weekly post-treatment infestations with *Ixodes ricinus*

**Fig. 5:**
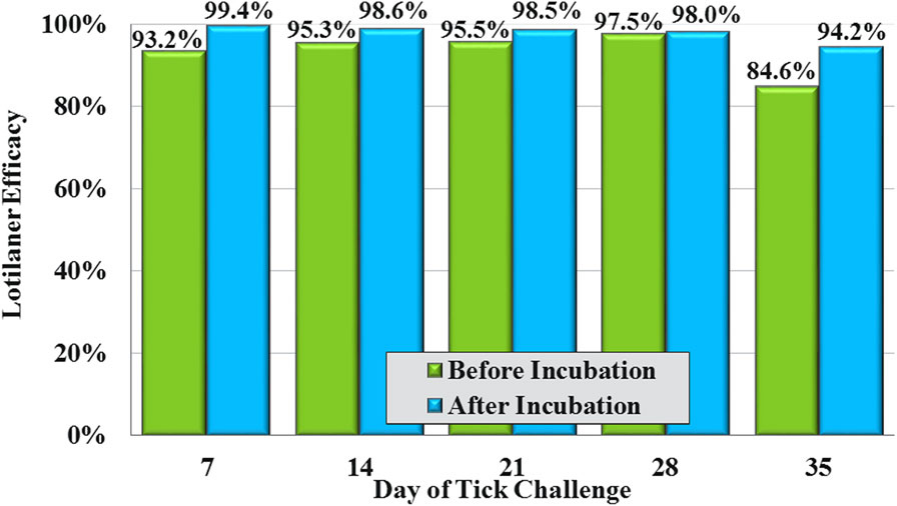
Percent reduction in geometric mean tick counts of lotilaner-treated dogs compared to untreated control groups at 12 h after weekly post-treatment infestations with *Ixodes ricinus*

Lotilaner treatment met the > 90% requirement to establish efficacy at all 8-h post-challenge assessments through Day 29 (Fig. 4). At all 12-h post-challenge assessments through Day 35, the reduction in mean tick counts compared to controls demonstrated the efficacy of lotilaner throughout the measured post-treatment period (Fig. 5). Across all post-treatment challenges, few live ticks were removed from lotilaner-treated dogs. Following incubation, 50% of these ticks showed no evidence of engorgement.

No abnormalities were detected in study dogs during clinical observations for behavior, salivation, pupil constriction, nervous signs and feces. There were three observations of adverse events in treated dogs: one dog had an incident of dark red feces with normal consistency, one had edema of the dermis due to a tick bite reaction and one dog had a superficial bite wound. None of these events were attributed to treatment.

## Discussion

The experimental methodology was validated, as at least 25% on average of the applied female *I. ricinus* ticks were attached to control dogs at each assessment. Additionally, the continued viability of the pre- and post-incubation ticks from these control groups, with no significant difference in live tick numbers before and after incubation, validated the incubation methodology in determining that the live ticks taken from lotilaner-treated dogs subsequently died as a result of the treatment. The rapid onset of lotilaner’s acaricidal activity in this study is consistent with the SOK demonstrated against fleas and can be attributed to the rapid achievement of maximum blood concentrations within 2 h of administration [11, 13]. As in laboratory studies using *Ctenocephalides felis* infestations, the high effectiveness of lotilaner in this study was maintained through 35 days when assessments of tick mortality were made at 12 h after each challenge.

Conclusions concerning between-product comparisons when studies are completed in different laboratories under different (albeit similar) conditions must be guarded. Nonetheless, the pre-incubation onset of tick SOK by lotilaner in this study appeared to match or compare favorably with reports from other isoxazoline studies (which did not include post-incubation effectiveness data). In our study, at 8 h post-treatment lotilaner pre-incubation efficacy against *I. ricinus* was 99.2%, while efficacies for sarolaner and fluralaner at this time point were 76.7 and 97.9%, respectively [18, 19]. For afoxolaner no data are available for the 8-h post-treatment time point, but at 12 h post-treatment it was found to be 93.4% effective against *I. ricinus* [20], implying a somewhat slower onset of activity than was observed for lotilaner in this study.

Reports of the post-treatment effectiveness of lotilaner provide support for its favorable sustained SOK relative to other isoxazolines. At the end of the labeled 1 month effectiveness period at 8 and 12 h post-challenge, mean tick count reductions for lotilaner (using pre-incubation results) were 75.9 and 97.5%, respectively. At these time points, following treatment with sarolaner, reductions in mean counts of *I. ricinus* compared to untreated controls were 23.2 and 94.9%, respectively [18]. For afoxolaner, 12 h post-challenge on Day 28, *I ricinus* tick count reductions were just 38.5% compared to an untreated control group [20].

Other studies have demonstrated that lotilaner was highly effective against *I. scapularis, D. variabilis* and *R. sanguineus* when assessments were completed at 48 h following challenges through 35 days after treatment, and against *A. americanum* when challenges were completed 28 days after treatment [12]. To date, there have been no reports of the SOK of any isoxazoline against *D. variabilis*, *R. sanguineus* and *A. americanum*, and it is hoped that further studies with these species can confirm that they are as susceptible as *I. ricinus* to lotilaner and to the other isoxazolines.

Transmission of pathogens such as *Borrelia burgdorferi*, *Anaplasma phagocytophilum*, *Rickettsia* and *Bartonella* species from tick to host typically appears to begin from 24 to 36 h after a tick begins to attach [14–16]. The possibility of earlier transmission cannot be completely discounted because of the extrusion of substances from a tick’s salivary glands during the process of attachment.

There is conflicting evidence as to whether systemically acting isoxazolines could be as effective as topically acting chemicals, which may have some repellent activity, in reducing the risk of tick-borne pathogen transmission. In one study, topical permethrin showed a significantly faster activity against ticks than afoxolaner and fluralaner [21]. Following challenge with *R. sanguineus*, there was no evidence of *Ehrlichia canis* transmission to permethrin-treated dogs, while transmission was demonstrated into four of eight dogs treated with afoxalaner and two of eight treated with fluralaner. The absence of evidence of *E. canis* transmission to the permethrin group was attributed to permethrin preventing tick attachment. That conclusion is in conflict with another study which found that throughout a month following treatment there were more live ticks on permethrin-treated than on sarolaner-treated dogs [22]. Regardless, the speed with which ticks are killed is important, and the faster the death of the tick, or at least the faster feeding is completely stopped, the lower the probability of disease transmission.

Isoxazolines have been shown to induce a paralysis in insect and acarine parasites through blockade of distinct binding sites on γ-aminobutyric acid- and glutamate-gated chloride channels [23]. In vitro, the onset of the neurological effects of isoxazolines on insects has been observed to occur within 10 min of exposure, progressing through incoordination to prostration [6]. Thus the actual time for insects and acarines to be classified as dead is longer than the time at which the paralyzing effects of the treatment occur. As an affected tick becomes moribund, these effects would likely interfere with engorgement and transmission of pathogens. Specific investigation is now needed to determine the degree to which lotilaner might interrupt or prevent such transmission.

The data presented in this paper indicate that lotilaner at least matches, and has the potential to exceed those of other tick control products in quickly killing ticks present at the time of treatment, and in providing a sustained rapid SOK throughout and beyond the monthly re-treatment period. As such, used as a product to control flea and tick infestations, lotilaner has potential as a safe and effective means of reducing the incidence of disease caused by tick-borne pathogens.

## Conclusion

Lotilaner administered orally to dogs at a minimum dose rate of 20 mg/kg began to kill *I. ricinus* ticks within 4 h of treatment and was 100% effective against existing infestations within 8 h. Relative to an untreated control group, lotilaner reduced mean *I. ricinus* live tick counts by 94.3% as soon as 8 h after challenges through 28 days after treatment. At 12 h after these infestations, lotilaner effectiveness of at least 94.2% was sustained through 35 days after treatment. Lotilaner has therefore been shown to be a valuable tool for achieving a rapid effect on existing tick infestations and for providing ongoing sustained rapid speed of kill in the four to 5 weeks following treatment. By quickly killing ticks that infest dogs, lotilaner has the potential to help limit the transmission of tick-borne pathogens.

## Additional file

Additional file 1: French translation of the Abstract. (PDF 47.5 KB)

## Abbreviations

ANOVA: Analysis of variance
GCP: Good clinical practice
SOK: Speed of kill
VICH: International cooperation on harmonization of technical requirements for registration of veterinary medicinal products
WAAVP: World association for the advancement of veterinary parasitology
