# Supplementary material for: Laboratory evaluation of the speed of kill of lotilaner (Credelio™) against Ixodes ricinus ticks on dogs
Source: Parasit Vectors. 2017 Nov 1;10:541. doi: 10.1186/s13071-017-2467-z (PMC5664588; doi:10.1186/s13071-017-2467-z)
Supplement: Additional file 1: — French translation of the Abstract. (PDF 47.5 KB) [file 13071_2017_2467_MOESM1_ESM.pdf]

Évaluation en laboratoire de la rapidité d'action du lotilaner (Credelio™) contre les tiques *Ixodes ricinus* chez le chien

Martin Murphy[1], Daniela Cavalleri[1], Wolfgang Seewald[1], Jason Drake[2]* et Steve Nanchen[1]

[1]Elanco Santé animale, Schwarzwaldallee 215, CH-4058 Bâle, WRO-1032.2.58, Suisse
[2]Elanco Santé animale, 2500 Innovation Way, Greenfield, IN 46140, États-Unis

*Correspondance : drake_jon_j@elanco.com

Adresse électronique :
Martin Murphy : murphy_martin_gerard@elanco.com
Daniela Cavalleri: cavalleri_daniela_a@elanco.com
Wolfgang Seewald : seewald_wolfgang@elanco.com
Jason Drake : drake_jon_j@elanco.com
Steve Nanchen : nanchen_steve@elanco.com

Résumé

Contexte : face à l'expansion géographique de certaines espèces de tiques et à la reconnaissance grandissante de leur rôle dans les maladies vectorielles, il existe un véritable besoin pour des solutions de contrôle sûres et rapidement efficaces chez le chien. Le lotilaner est un nouvel agent de la classes de isoxazolines disponible sous forme de comprimés à croquer aromatisés (Credelio™). Après administration, il est rapidement absorbé et présente une efficacité d'au moins 98 %, pendant au moins un mois, comme le montrent les évaluations réalisées 48 heures après le traitement et suite à de nouvelles infestations. Une étude a été conduite afin de déterminer la rapidité avec laquelle le lotilaner tue les tiques.

Méthodes : à J-4, 38 chiens ont été infestés par *Ixodes ricinus*. Parmi eux, les 32 chiens présentant les nombres les plus élevés de tiques ont été randomisés dans quatre groupes : deux groupes témoins, non traités, et deux groupes recevant des comprimés de lotilaner à une dose minimale de 20 mg/kg. Les chiens ont été infestés par *I. ricinus* à J-2, J7, J14, J21, J28 et J35. Des comptages ont été effectués 4 et 8 heures après le traitement (J0) et 8 et 12 heures après les infestations suivantes.

Toutes les tiques vivantes ont été maintenues dans un incubateur pendant 24 heures après leur récolte sur les chiens de l'étude.

Résultats : à 4 heures du traitement, une réduction de 69,8 % de la moyenne géométrique du nombre de tiques vivantes a été observée chez les chiens traités par rapport aux témoins. Après incubation, la diminution a atteint 97,2 %. A 8 heures du traitement, les diminutions avant et après incubation ont respectivement été de 99,2 % et 100 %. Suite aux nouvelles infestations réalisées après le traitement, l'efficacité à 8 et 12 heures après incubation a été respectivement d'au moins 94,3 % et 98,0 % jusqu'à J28, puis de 85,7 % et 94,2 % à 8 et 12 heures après l'infestation de J35. Le nombre moyen de tiques vivantes était significativement inférieur dans les groupes lotilaner par rapport aux groupes témoins lors de toutes les évaluations réalisées jusqu'à J35 à 8 ($t_{(7)} \geq 9$, $p < 0{,}0001$, J0 à J28 ; $t_{(7)} = 3{,}54$, $p < 0{,}0095$, J35) et 12 heures du traitement et après les infestations suivantes ($t_{(7)} \geq 10$, $p < 0{,}0001$, tous les jours). Aucun événement indésirable lié au traitement n'a été observé.

Conclusion : le lotilaner, administré à une dose minimale de 20 mg/kg, a commencé à montrer un effet acaricide contre les tiques infestant les chiens dans les quatre heures suivant le traitement et a atteint une efficacité de 100 % dans les 8 heures. Une persistance de cet effet létal rapide a été observée suite aux nouvelles infestations par *I. ricinus* tout au long des 35 jours. Grâce à son action rapide sur les tiques infestant le chien, le lotilaner peut potentiellement contribuer à limiter la transmission vectorielle de pathogènes par les tiques.
